# Multi-Label Classification in Anime Illustrations Based on Hierarchical Attribute Relationships

**DOI:** 10.3390/s23104798

**Published:** 2023-05-16

**Authors:** Ziwen Lan, Keisuke Maeda, Takahiro Ogawa, Miki Haseyama

**Affiliations:** 1 Graduate School of Information Science and Technology, Hokkaido University, N-14, W-9, Kita-ku, Sapporo 060-0814, Hokkaido, Japan; lan@lmd.ist.hokudai.ac.jp; 2 Faculty of Information Science and Technology, Hokkaido University, N-14, W-9, Kita-ku, Sapporo 060-0814, Hokkaido, Japan; maeda@lmd.ist.hokudai.ac.jp (K.M.); ogawa@lmd.ist.hokudai.ac.jp (T.O.)

**Keywords:** hierarchical classification, anime illustration, attribute classification, graph convolutional networks, generative adversarial networks

## Abstract

In this paper, we propose a hierarchical multi-modal multi-label attribute classification model for anime illustrations using a graph convolutional network (GCN). Our focus is on the challenging task of multi-label attribute classification, which requires capturing subtle features intentionally highlighted by creators of anime illustrations. To address the hierarchical nature of these attributes, we leverage hierarchical clustering and hierarchical label assignments to organize the attribute information into a hierarchical feature. The proposed GCN-based model effectively utilizes this hierarchical feature to achieve high accuracy in multi-label attribute classification. The contributions of the proposed method are as follows. Firstly, we introduce GCN to the multi-label attribute classification task of anime illustrations, enabling the capturing of more comprehensive relationships between attributes from their co-occurrence. Secondly, we capture subordinate relationships among the attributes by adopting hierarchical clustering and hierarchical label assignment. Lastly, we construct a hierarchical structure of attributes that appear more frequently in anime illustrations based on certain rules derived from previous studies, which helps to reflect the relationships between different attributes. The experimental results on multiple datasets show that the proposed method is effective and extensible by comparing it with some existing methods, including the state-of-the-art method.

## 1. Introduction

Recently, the anime industry has experienced significant growth, leading to an increase in research on anime illustrations. Various studies, such as illustration editing [1,2], illustration super-resolution [3], cartoon face generation [4,5], and line art colorization [6,7] have been conducted. As the number and complexity of anime illustrations continue to grow, there is a growing need for classification techniques. Efficient management and the classification of numerous illustrations are critical for creators. Automated identification and organization of specific elements within images through the classification techniques for anime illustrations can significantly streamline the animation production process. Developing effective classification techniques requires a thorough understanding of the contents of these illustrations.

In computer vision, image classification is an important task, which involves analyzing and understanding visual data from the environment using computers. Multi-label image classification, a variation of image classification, allows for multiple labels or tags to be assigned to an image. This is different from traditional image classification, which involves assigning a single label or class to an image. Multi-label image classification is useful in cases where an image contains multiple objects or features relevant to the task. For instance, medical image classification [8] involves identifying and labeling multiple organs or structures in an X-ray image. Similarly, in recommendation systems [9], an image of a product may contain multiple features relevant to the user, such as color, size, and material.

In multi-label image classification, it is important to consider the relationships between labels to enhance classification accuracy. This is because objects in images frequently co-occur, appearing together in the same image. Graph convolutional networks (GCNs) [10] are neural networks that can predict relationships between labels on graph structures. They are particularly useful in multi-label image classification because they can capture the dependencies between different labels and use this information to improve classification accuracy. For example, Chen et al. proposed a GCN-based multi-label image classification system that used a complete graph to model the correlation between various labels [11]. This approach was effective in enhancing classification accuracy. GCN-based approaches are likely to be highly effective for anime illustrations because objects in amine illustrations frequently co-occur as well. Anime illustrations typically describe complex scenes with many elements, and GCNs can help us understand the relationships between these objects and improve classification accuracy.

Distinguishing anime illustrations from real-world images requires a different approach to image classification. Anime illustrations are artificially produced and typically exhibit unique characteristics that are absent in real-world images. These illustrations often feature stylized objects or characters with exaggerated or uncommon attributes that are critical for precise classification. In addition to simple objects, it is necessary to consider the attributes of these objects when building classification methods for anime illustrations. For example, if we are classifying an illustration of an anime character, we may need to consider not only the object (e.g., character) but also the attributes of that object (e.g., hair color and eye shape). To the best of our knowledge, no research has investigated the multi-label attribute classification task [12,13] for anime illustrations.

In previous GCN-based multi-label classification methods [11,14], labels were typically treated equally when constructing graphs to model co-occurrence relationships between them. This is appropriate since these labels represent “objects” rather than “attributes.” However, attributes have a clear hierarchy with upper and lower inclusion levels indicating semantic relationships of subordination between parent and child labels. Therefore, to utilize GCN-based multi-label classification methods for attribute classification in anime illustrations, it is essential to consider the hierarchy of attributes.

In this paper, we propose multi-label image classification in animation illustration with GCNs, considering hierarchical relationships of attributes. The proposed method consists of three key operations: hierarchical divisive clustering (HDC), hierarchical label assignment (HLA), and GCN-based classification. We extract categorized feature representations from anime illustrations in HDC and integrate the representations according to a pre-defined hierarchical label structure to obtain a feature containing rich hierarchical relationships between labels in HLA. Specifically, for the HDC part, we obtain feature representations of the anime illustrations belonging to different categories by divisive clustering. Inspired by the previous study [15], we use a clustering algorithm based on multiple generative adversarial networks (GANs) organized in a binary tree structure, which can obtain more appropriate category representations on datasets with a variety of styles, just as with the anime illustration dataset. In the HLA part, following the pre-defined hierarchical label structure from the dataset, the obtained feature representations are organized by hierarchical label assignments to form a feature with rich hierarchical relationships between labels. Additionally, because the general anime illustration datasets do not exclude a defined attribute hierarchy, we use WordNet [16] to construct the attribute hierarchy in the anime illustration datasets based on the logical relationships between words. Finally, the feature with rich hierarchical relationships is inputted into GCN for classification, which considers the hierarchy of attributes, and can improve the overall accuracy of the model.

In summary, the contribution of this study can be highlighted as follows:

- We propose a GCN-based model for the multi-label attribute classification suitable for anime illustrations.
- Considering the hierarchical relationships between attributes, we use hierarchical clustering and organize the attribute representations of anime illustrations by hierarchical label assignments to generate a feature with rich hierarchical relationships between labels that can be imported into the GCN-based classification model.
- We construct a hierarchical structure of attributes in the anime illustration datasets based on the defined logical relationships between words, which helps better reflect the relationships between different attributes in the classification process.

## 2. Related Works

In this section, related works are briefly reviewed in the following three categories.

### 2.1. Attribute Classification

The attribute classification task involves identifying the descriptive properties (or attributes) of objects in images. Because it requires a deep understanding of the features of the object in the target image, it is a more complex task than simply classifying the objects themselves. It has been a topic of interest in computer vision for a long time, as it has many practical applications. For example, attribute classification can improve image search engines by allowing users to search for images based on specific attributes rather than just objects. Additionally, it can enhance image recommendation systems by suggesting images based on the attributes of the objects they depict.

In attribute classification tasks, it is common for certain attributes to be correlated with each other. For instance, the attribute *beard* is typically found in conjunction with the attribute *male*. In other words, if an image contains the attribute *beard*, it is more likely also to contain the attribute *male*. Several approaches have been used to address the issue of correlated attributes in attribute classification tasks. Some studies [13,17] have ignored the correlations between attributes and learned them independently, whereas others [18,19,20,21] have used a multi-task learning approach that explicitly models the correlations between attributes. The latter approach involves training a classifier to predict multiple attributes, with the assumption that predicting one attribute enhances the probability of predicting related attributes. It has been demonstrated that models considering the correlations between attributes tend to perform better in classification tasks than models that cannot consider them.

Our approach to addressing attribute correlations was inspired by the study [13]. Although the study did not specify how to utilize attribute correlations, it provided a semantic interpretation of attributes. In this study, attributes were classified hierarchically according to semantic information, which contains low-level visual adjectives (e.g., *color*, *shape*), inherent object features (e.g., *material*), and high-level object components (e.g., *having a tail* and *wearing sunglasses*). This hierarchical structure enables a more nuanced understanding of the relationships between attributes and can improve the classifier’s performance. Since attribute correlations are present not only in real-world images but also in anime illustrations, we expect that the hierarchical structure approach will be effective in classifying the attributes of anime illustrations as well.

### 2.2. Hierarchical Classification

The use of hierarchical structures in multi-label classification tasks provides valuable insight into label relationships. In a previous study [22], the researchers categorized hierarchical multi-label learning methods into two categories: local and global models. These approaches aim to capture structural relationships between labels in different ways.

The local model in hierarchical multi-label classification involves constructing multiple classifiers within the hierarchy and aggregating their results to obtain an overall classification for the entire label space. It allows for incorporating additional fine-grained hierarchical information and can be useful in situations with strong dependencies between labels. For example, the researchers proposed a top-down hierarchical multi-label classification method [23] using a hierarchical support vector machine, which only applies to a node if its parent labels are positive. This local model can use additional fine-grained hierarchical information. However, this model is susceptible to error propagation and frequently requires the construction of multiple classification modules when building it.

On the other hand, the global model typically consists of a single classification module that directly uses hierarchical structure information. It can incorporate global relationships between labels and can be more efficient than the local model, as it does not require the construction of multiple classification modules. For example, some global models [24] use the hierarchy to construct recursive regularization loss terms to constrain classification parameters. This approach uses the relationships between labels in the hierarchy to regularize the model and prevent overfitting. Furthermore, as the hierarchical multi-label classification task corresponds to the relationships among labels stored in the hierarchy, an increasing number of studies are considering not only the information provided by the classification target but also the corresponding representation of the hierarchical structure of labels [25,26]. These methods assign varying weights to distinct parts of the content representation that are the most associated with each label in the hierarchy, taking into account the interdependence between the representation of the hierarchical structure and the classification target. This approach is suitable for our method.

### 2.3. Clustering Based on the Generative Adversarial Network (GAN)

Clustering is a common unsupervised learning technique used in various computer vision tasks to obtain excellent image category representations. In recent years, with the development of GAN [27], this model has achieved significant success in many unsupervised learning tasks, and the clustering task is certainly no exception. GAN can easily capture the underlying data distribution from a given set of samples by defining a mapping from a predefined latent before the target distribution. However, earlier versions of GANs suffer from overfitting and mode-collapse issues due to the imbalance between the discriminator and the generator [28]. To overcome the above weaknesses, various methods have been proposed, including unrolled GAN [29], which introduces a surrogate objective function that simulates a discriminator response to generator changes, VEEGAN [30], which casts implicit probability distributions to minimize the joint distribution, and MGGAN [31], which develops a manifold space by the pre-trained autoencoder to reconstruct all of the samples. Although these approaches address the issues of overfitting and mode collapse, they are unable to meet the requirements of multi-label classification with a single prior and the capacity of a single generator transformation. Recently, researchers have introduced a tree structure, called the hierarchical GAN-Tree [15], to facilitate the clustering by a multi-generator mode. This method can be utilized together with the corresponding prior distribution to generate samples with the desired level of quality and diversity. Training multiple GANs for different data will be an appropriate way to tackle the combination of multiple labels and the hierarchical structure.

## 3. Proposed Method

**Figure 1:**
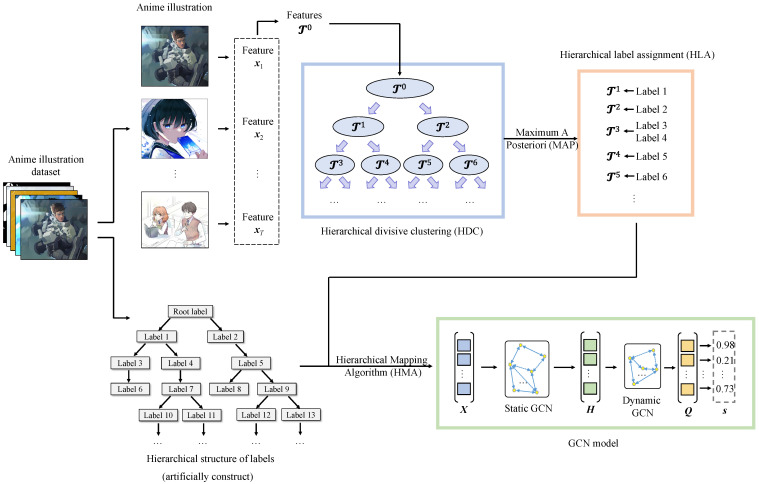
Overview of the proposed method. It consists of three core operations: hierarchical divisive clustering (HDC), hierarchical label assignment (HLA), and GCN-based classification. We extract categorized feature representations from anime illustrations in HDC, and integrate the representations according to a pre-defined hierarchical label structure to obtain a feature with rich hierarchical relationships between labels in HLA that can be imported into the GCN model for the final classification.

Figure 1 shows the architecture of the proposed method. As shown in Figure 1, the proposed method consists of three core parts: HDC, HLA, and GCN-based classification. We will explain these three parts in detail in the following subsections.

### 3.1. Hierarchical Divisive Clustering (HDC)

In this subsection, we will explain the hierarchical divisive clustering (HDC) details. Specifically, in HDC, we adopt the hierarchical GAN-Tree [15] mentioned in Section 2.3, which is used to perform the clustering. A hierarchical GAN-Tree is a hierarchical feature representation that transforms the original feature-embedding into a full binary tree. In detail, it continues to split samples into two different clusters based on the most discriminative feature difference obeying the target distribution with multiple GANs. We apply the adversarially learned inference [32] framework as the basic GAN formulation for the hierarchical GAN-Tree in practice, which enables the generation of plausible samples from the predefined latent distributions.

**Figure 2:**
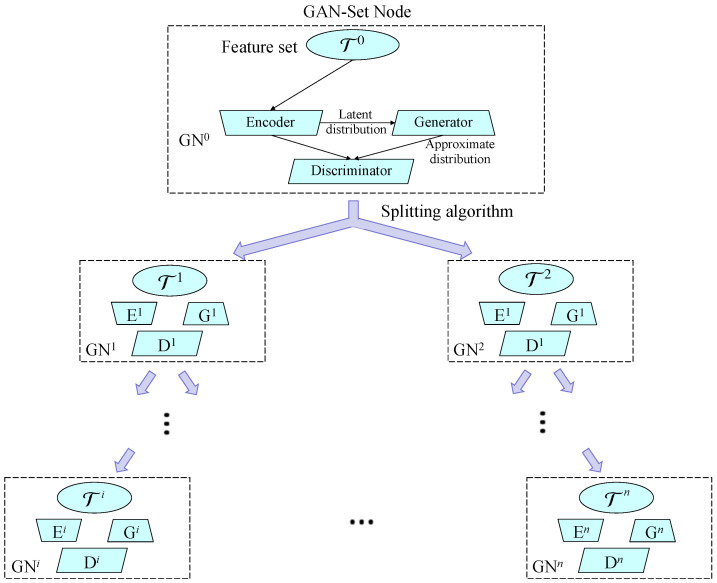
Outline of the hierarchical GAN-Tree architecture. The composition of a single GAN-Set node at the root level shows how the networks are used.

The clustering target of the hierarchical GAN-Tree is the feature set $T^{0}={\left\{x_{t}\right\}}_{t=1}^{T}$ composed of features extracted from images t(t=1,2,...,T) in the original dataset. Without considering the number of labels in each level, the hierarchical GAN-Tree plans to split each parent sample into two children clusters by GAN-Set node $GN^{i}$. GAN-Set node $GN^{i}$ is an individual GAN framework, which includes an encoder $E^{i}$, a generator $G^{i}$, and a discriminator $D^{i}$, as shown in Figure 2. The input of each $GN^{i}$ is the target feature set $T^{p}$, which is drawn from the real image sample distribution $P_{d}$ of its parent node (we assume *p* to be the parent node index of the child node *i*). $GN^{i}$ is trained to look for and output the best possible approximate distribution $P_{g}^{i}$ of the target data distribution $P_{d}$. During the training process, the approximation is improved by the latent distribution $P_{Z}^{i}$ in the succeeding hierarchy of the hierarchical GAN-Tree. The latent distribution $P_{Z}^{i}$ is derived from the latent prior vector $z_{t}\in Z$ corresponding to feature $x_{t}$, where Z is the latent space following the prior distribution (Gaussian distribution).

To avoid the mode-collapse problem caused by the unstable generator $G^{i}$, hierarchical GAN-Tree employs the splitting algorithm to exploit the highly discriminative features embedded in the image. The splitting algorithm aims to form two mutually exclusive and collectively exhaustive target data clusters, by utilizing the likelihood of the latent representations to the predefined prior distributions. This algorithm is based on two types of losses, i.e., $L_{nll}$ and $L_{recon}$. $L_{nll}$ is used to maximize the utilization of the likelihood of latent representations to the prior distributions, while $L_{recon}$ is used as a regularization to hold the semantic uniqueness of the individual samples in the split clusters. The details of $L_{nll}$ and $L_{recon}$ are defined as follows:

(1) $$\begin{array}{cc} L_{nll} & =\frac{1}{T_{i}}\sum_{t=1}^{T_{i}}-\log p\left(z_{t}\right), \end{array}$$

(2) $$\begin{array}{cc} L_{recon} & =\frac{1}{T_{i}}\sum_{t=1}^{T_{i}}{∥x_{t}-G^{i}\left(z_{t}\right)∥}_{2}^{2}. \end{array}$$

By optimization of the final splitting loss function $L_{split}=L_{nll}+L_{recon}$ using the Adam optimizer [33], the splitting algorithm obtains the hard-assigned label from the target parent samples for the left or right child samples. In advance, the hierarchical GAN-Tree uses a robust stopping criterion based on the information change rate (ICR) [34] to guarantee that the hierarchical GAN-Tree avoids overfitting to the target data samples. So far, we have obtained feature representations of the anime illustrations belonging to different categories by divisive clustering.

### 3.2. Hierarchical Label Assignment (HLA)

In this subsection, we explain the details of the hierarchical label assignment (HLA). In the previous phase, we transformed the original features from images into a full binary tree structure composed of $GN^{i}$. Each $GN^{i}$ includes a cluster $T^{i}$ that is awaiting allocation with relevant hierarchical labels. Therefore, we must assign suitable hierarchical labels for each feature set $T^{i}$. Since our study is dedicated to solving the multi-label classification task, multiple labels may be assigned to the same feature. We define the feature $x_{t}$ tagged by the hierarchical label *v* as $x_{t}^{\left(v\right)}$.

We use maximum a posteriori (MAP) [35] to obtain the optimal potential label through estimating the mapping from the feature $x_{t}^{\left(v\right)}$ distribution to the latent distribution $P_{Z}^{i}$. The maximum posterior probability $\rho_{max}^{i}$ of the uncategorized feature set $T^{i}$ is defined as follows:

(3) $$\begin{array}{cc} \rho_{max}^{i} & =max_{v}\prod_{t=1}^{sum}p\left(v|t\subset P_{g}^{i}\right)\\ & =min_{v}[\sum_{t=1}^{sum}p\left(v|E^{i}\left(x_{t}^{\left(v\right)}\right)\right)-P_{Z}^{i}]. \end{array}$$

When the maximum posterior probability $\rho_{max}^{i}$ is calculated, we can set the probability interval to obtain the remaining potential hierarchical labels for the uncategorized feature set $T^{i}$. If the posterior probability satisfies the interval $\left[\rho_{max}^{i}-\delta ,\rho_{max}^{i}\right]$, the corresponding hierarchical label *v* is a potential label assigned to the set $T^{i}$. We set δ=0.02 in our study, avoiding excessive labels for each uncategorized feature set.

**Figure 3:**
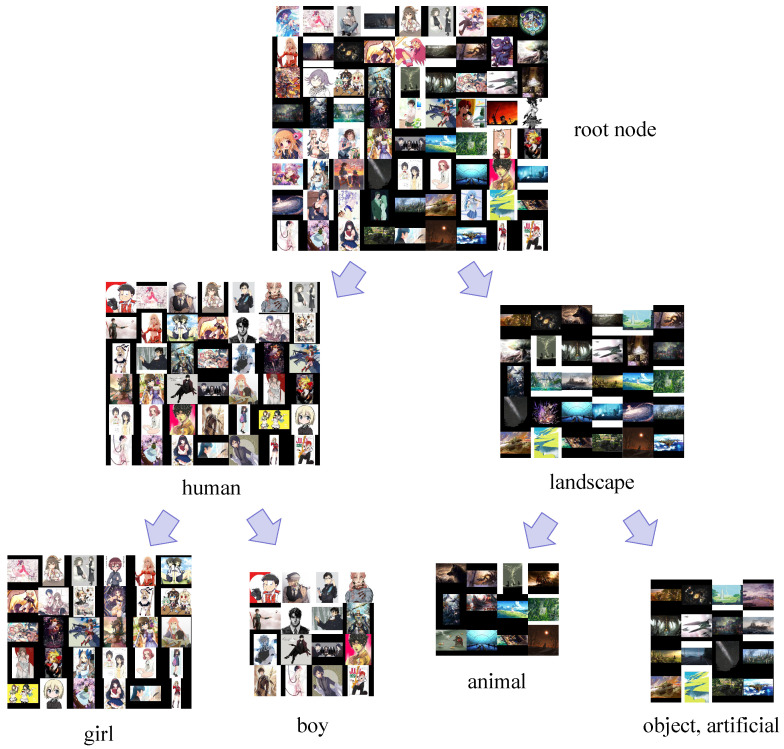
Clustering and label assigning in the HDC and HLA procedure on the Safebooru dataset [36].

For each potential hierarchical label *v*, it will become the suitable label for $T^{i}$ when the following two conditions are satisfied. First, the tagged sample $x_{t}^{\left(v\right)}$ has to satisfy the following condition:

(4) $$\begin{array}{c} max_{t\in T^{i}}p\left(E^{i}\left(x_{t}^{\left(v\right)}\right)\right)>ICR, \end{array}$$

where ICR [34] is a probability measure to handle the average log-likelihood for the whole feature set $T^{i}$. This precondition guarantees that the sample $x_{t}^{\left(v\right)}$ is qualified as the representation of the cluster $T^{i}$. Second, the parent and ancestor labels of the hierarchical label *v* will not be present in the subsets of $T^{i}$. If a hierarchical label *v* is confirmed to be assigned to $T^{i}$, this hierarchical label will no longer be suitable to the other uncategorized feature cluster. Hence, we assign each $T^{i}$ of $GN^{i}$ with several suitable labels in a top-down pattern. The most discriminative semantic differences mainly cause highly discriminative feature differences, which means that the high-level labels will be more suitable for the top feature cluster in the binary tree than the low-level ones. The hierarchical GAN-Tree first split the highly discriminative feature cluster from the whole sample based on the splitting algorithm. Therefore, the HLA attempts to deploy the label assignment in a semantic coarse-to-fine pattern corresponding to the top-down traversal of the feature cluster in the binary tree. Figure 3 shows how some of the anime illustrations in a common dataset, Safebooru [36], are clustered and assigned labels after the HDC and HLA procedures.

To obtain the feature representations of anime illustrations that contain hierarchical relationships between labels and can be imported into the GCN for final classification, we adopt the hierarchical mapping algorithm (HMA) module proposed in a previous study [25]. The HMA module assigns weights $W_{h}$ to different parts of the feature representation using the content most associated with each label in the hierarchy. This enables us to obtain a hierarchical feature representation that is most suitable for the task. First, we embed the given hierarchical label structure into a randomly initialized matrix $S\in R^{C\times d_{a}}$, which represents the embedding of the hierarchical category with the $d_{a}$-dimension. *C* represents the number of categories. For the feature $x_{t}\in R^{N\times D}$ extracted from the image *t*, we perform the following calculation to obtain the weights $W_{h}\in R^{C\times N}$:

(5) $$\begin{array}{c} W_{h}=softmax\left(A_{h}S\cdot \tanh\left(W_{s}x_{t}^{⊤}\right)\right), \end{array}$$

where $A_{h}\in R^{C\times C}$ represents the correlation matrix of $x_{t}$ based on the assigned labels in the given label hierarchy, $W_{s}\in R^{d_{a}\times D}$ denotes a randomly initialized weight matrix, $d_{a}$ is a hyperparameter that we can arbitrarily set, and the softmax(·) function ensures that all of the computed weights sum up to 1 for each category. After that, we obtain the feature representations of anime illustrations that contain hierarchical relationships between labels, denoted as $H\in R^{C\times D}$, by the following equation:

(6) $$\begin{array}{c} H=W_{h}x_{t}. \end{array}$$

In this way, the feature H with rich hierarchical relationships between labels is obtained.

### 3.3. GCN-Based Classification

In this subsection, we apply H sequentially into a static GCN and a dynamic GCN to obtain different representations of the label relations for specific input images for the final classification. We first feed H into a single-layer static GCN. The output of the static GCN, denoted as $V\in R^{C\times D}$, is defined as follows:

(7) $$\begin{array}{c} V=LReLU(A_{st}{\mathrm{HW}}_{st}), \end{array}$$

where $A_{st}$ represents the correlation matrix, and $W_{st}$ represents the state update weights. LReLU(·) denotes the activation function LeakyReLU [37], which is a variant of the standard ReLU function that allows a small number of negative values to pass through. This is useful for preventing the model from being stuck in a state where all the neurons are deactivated, which can occur with standard ReLU. After that, V is then input into the dynamic GCN. The output of the dynamic GCN, denoted as $Q\in R^{C\times D}$, is calculated using the following equation:

(8) $$\begin{array}{c} Q=LReLU(A_{dy}{VW}_{dy}), \end{array}$$

where LReLU(·) denotes the LeakyReLU activation function, $A_{dy}$ denotes the dynamic correlation matrix, and $W_{dy}$ denotes the state update weights. $A_{dy}$ is calculated using a conv layer with weights $W_{A}$ applied to $V^{\prime }$, followed by the sigmoid activation function σ(·) as $A_{dy}=\sigma \left(W_{A}V^{\prime }\right)$. As a result, this GCN flow can capture the co-occurrence between different attributes in the illustration while utilizing the hierarchical relationships between them, which can improve classification accuracy.

We will now describe the process of the final classification and the calculation of the loss function. The output of the dynamic GCN, $Q={\left[q_{1},q_{2},\cdots ,q_{C}\right]}^{⊤}$, is used for the final classification. Specifically, we input each vector $q_{c}$ of the final category representation Q into a fully connected layer to obtain the predicted scores $s^{c}$ for category *c*. These scores are then concatenated to form the final score vector $s={\left[s^{1},s^{2},\cdots ,s^{C}\right]}^{⊤}$. According to the previous works [11,14,38,39], the loss function $L_{G}\left(y,s\right)$ can be defined as follows:

(9) $$\begin{array}{c} L_{G}\left(y,s\right)=\sum_{c=1}^{C}y^{c}\log\left(\sigma \left(s^{c}\right)\right)+\left(1-y^{c}\right)\log\left(1-\sigma \left(s^{c}\right)\right), \end{array}$$

where $y\in R^{C}$ represents the ground truth labels for an image, and $y^{c}=\left\{0,1\right\}$ indicates whether label *c* is present or absent in the image.

## 4. Comparison Experiments

In this section, we present experimental results to demonstrate the effectiveness of our proposed method. In Section 4.1, we introduce the anime illustration datasets used in the experiment and explain how we constructed the hierarchical structure for the labels in the datasets. In Section 4.2, we describe the experimental conditions and the implementation details. In Section 4.3, we introduce this experiment’s comparison methods and evaluation metrics used in this experiment. Finally, in Section 4.4, we present the experimental results.

### 4.1. Anime Illustration Datasets and Construction of Label Hierarchy

To verify the effectiveness and scalability of the proposed method, we used the following four datasets of anime illustrations to perform the experiments.

- **Safebooru** [36]: The Safebooru dataset is a comprehensive anime illustration dataset with over 1.0 million illustrations and 30 million labels. It is a subset of the Danbooru dataset, the largest dataset in the field of anime illustration, where illustrations tend to be non-pornographic and non-violent, and each illustration is accompanied by metadata, such as content labels and the names of the artists. We randomly selected 25,000 anime illustrations from the dataset, of which 75% were used as the training set and 25% as the test set, following the division of the original dataset.
- **DAF:re** [40]: The DAF:re (DanbooruAnimeFaces:revamped) dataset is a crowd-sourced, long-tailed dataset with almost 50,000 images spread across more than 3000 classes. It is also a subset of the Danbooru dataset, and is mainly used for animated character recognition, but unlike the usual dataset for character recognition, each image in this dataset is labeled with attributes other than the label indicating the character names. According to the description by the authors of this dataset in [40], the proportion of images in the training set, validation set, and test set are 70%, 10%, and 20%, respectively.
- **FG-BG** [41]: The FG-BG dataset is a dataset of anime illustrations used for character background segmentation. It consists of 18,500 illustrations from the Danbooru dataset, including illustrations with transparent backgrounds that only contain characters, illustrations with pure backgrounds that do not contain characters, and ordinary illustrations with characters and backgrounds. Following the previous study [41], we divided this dataset into a training set containing 75% of the images and a test set containing 25% of the images, and it should be noted that the proportions of the three types of images mentioned above in the subset are the same as the whole dataset.
- **iCartoonFace** [42]: The iCartoonFace is a benchmark dataset of 389,678 images of 5013 characters annotated with character names and other auxiliary attributes. In character recognition of anime illustrations, this dataset is exceptional due to its large-scale nature, high quality, rich annotations, and coverage of multiple occurrences, including near-duplications, occlusions, and appearance changes. The difference with the DAF:re dataset, which is also used for character recognition, is that this dataset is not a subset of the Danbooru dataset. In our experiments, we randomly selected 25,000 anime illustrations from the dataset, of which 75% were used as the training set and 25% as the test set following the division of the original dataset.

To construct accurate and convincing hierarchical relations for the labels in the datasets, we used the semantic relations of words defined in a large dictionary, WordNet [16], where words are grouped into a hierarchy defined by superordinate and subordinate relations. To select labels from the datasets for classification, we first filtered out the 500 most frequently appearing labels. Because some of these 500 labels are difficult to classify into a semantic hierarchy, we must remove them and use the remaining tags to build the hierarchy. In our experiment, the following labels are removed.

- Labels of the title of the work, the name of the character, the name of the illustrator, etc. (e.g., *hatsune miku*).
- Labels describing information about the illustration, not the content of the illustration (e.g., *absurdres*).
- Labels describing the art style to which the illustration belongs (e.g., *traditional media*, *monochrome*, *sketch*).
- Labels describing the character’s facial expression, movement, or pose (e.g., *happy*, *standing*).
- Labels describing the layout of the illustration (e.g., *upper body*, *cowboy shot*).

**Figure 4:**
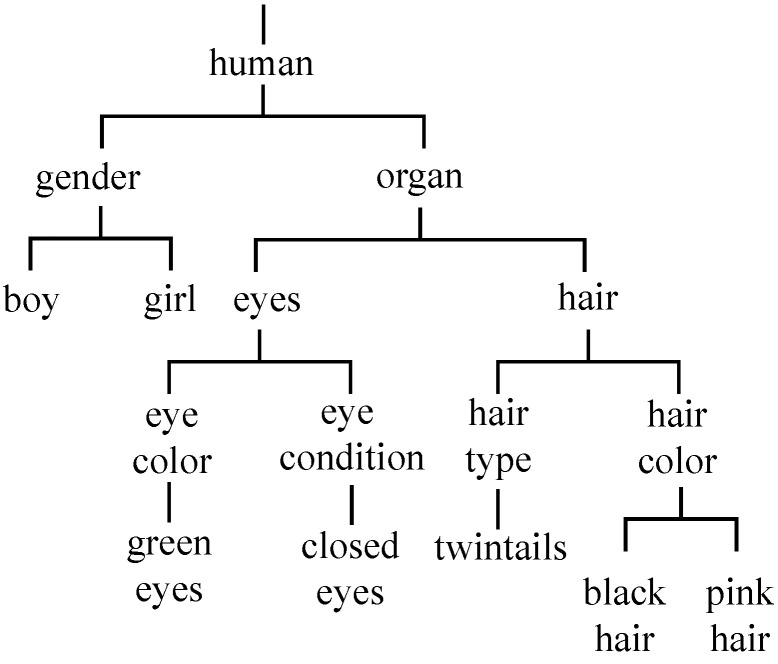
Part of the label hierarchy of the anime illustration datasets.

After removing the above labels, the remaining 359 labels were matched with words in WordNet [16] to construct the hierarchical relationship. Finally, seven layers of the hierarchical structure were constructed. Figure 4 shows a part of this hierarchy. As depicted in the figure, the directly connected parent and child nodes are labeled with an inclusive relationship.

### 4.2. Experimental Conditions and Implementation Details

We used ResNet-101 [43] as the backbone of the GCN-based attribute classification model. The negative slope of the LeakyReLU activation function, which was used in the GCN module, was set to 0.2. To improve the model’s generalization ability of the model, we used data augmentation techniques on the input images. We randomly cropped the images, resized them to 448×448 pixels, and horizontally flipped them, which can artificially increase the size of the training set by creating new images from the existing ones and help the model learn to be more robust to variations in the input data. To optimize the model, we used stochastic gradient descent as the optimizer. We set the momentum decay to 0.9 and the weight decay to $1.0\times 10^{-4}$. The learning rates for the different modules of the model were initially set at 0.5 for the GCN module, and 0.05 for the backbone CNN. Additionally, for our hierarchical GAN-Tree, we follow the DCGAN setting [44] for the generator, discriminator, and encoder networks, i.e., 56 × 56 with a prior multi-generator function [15] for all of the datasets.

### 4.3. Comparison Methods and Evaluation Metrics

To evaluate the effectiveness of the proposed method for multi-label attribute classification of anime illustrations, we compared it with several other methods. These methods were as follows.

- **ResNet-101** [43], an extensively used CNN for image classification tasks.
- **DAN** [12], a method that uses CNNs to learn discriminative features for multi-label attribute classification on real-world images.
- **SSGRL** [45], a method that uses CNNs and a graph propagation mechanism to improve the multi-label classification performance.
- **ML-GCN** [11], a method that uses GCNs to model the correlations between labels in the multi-label classification task.
- **ADD-GCN** [14], a method that constructs dynamic graphs to describe label relationships in images and uses an attention mechanism in the feature extraction to improve the GCN-based multi-label classification performance.
- **DSGCN** [39], a method that uses domain-specific semantic features from the image in the multi-label classification task for anime illustrations.
- **P-GCN** [38], a state-of-the-art method that uses a GCN to improv the multi-label classification performance, which is an extended version of ML-GCN.

The proposed method used ResNet-101 as its backbone, and these methods were trained using similar hyperparameters for a fair comparison. We evaluated the performance of each method on multi-label attribute classification for anime illustrations and compared their results with the proposed method to verify its effectiveness.

We adopted the following evaluation metrics following previous studies [11,14,38,39]: Averages of overall precision (OP), recall (OR), and F1 score (OF1); averages of per-class precision (CP), recall (CR), and F1 score (CF1). These metrics are calculated as follows:

(10) $$\begin{array}{cc} \mathrm{OP} & =\frac{\sum_{k}N_{k}^{c}}{\sum_{k}N_{k}^{p}},\;\;\;\;\mathrm{CP}=\frac{1}{C}\sum_{k}^{}\frac{N_{k}^{c}}{N_{k}^{p}}, \end{array}$$

(11) $$\begin{array}{cc} \mathrm{OR} & =\frac{\sum_{k}N_{k}^{c}}{\sum_{k}N_{k}^{g}},\;\;\;\;\mathrm{CR}=\frac{1}{C}\sum_{k}^{}\frac{N_{k}^{c}}{N_{k}^{g}}, \end{array}$$

(12) $$\begin{array}{cc} \mathrm{OF}1 & =\frac{2\times \mathrm{OP}\times \mathrm{OR}}{\mathrm{OP}+\mathrm{OR}},\;\mathrm{CF}1=\frac{2\times \mathrm{CP}\times \mathrm{CR}}{\mathrm{CP}+\mathrm{CR}}, \end{array}$$

where *C* is the number of classes, $N_{k}^{p}$ is the number of retrieved images for the *k*-th label, $N_{k}^{c}$ is the number of images that are correctly retrieved for the *k*-th label, $N_{k}^{g}$ is the number of ground truth images for the *k*-th label.

**Figure 5:**
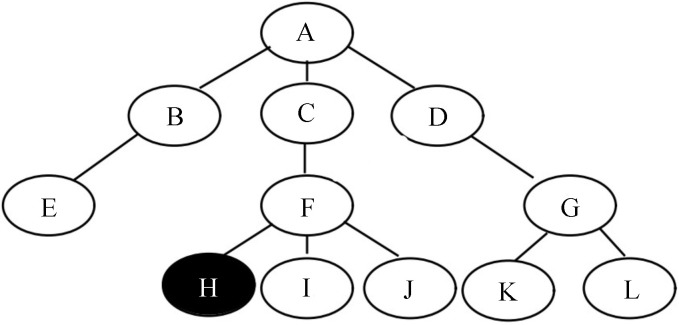
An example of the hierarchical structure of labels, where *H* is the ground truth of the image.

The metrics above consider only the final leaf node predictions and ignore the hierarchy of labels. It means that all leaf nodes are treated as equal without any special treatment of the different relationships of the nodes in the hierarchy. To emphasize the hierarchical nature of the labels, we do not expect classification errors at different parts of the hierarchy to be penalized in the same way. Therefore, we used hierarchical precision (HP), hierarchical recall (HR), and the hierarchical F1 score, (HF1) following previous studies [46,47]. First, let $C_{m}^{g}$ be the set of ground truth labels and $C_{m}^{p}$ be the set of predictive labels for image *m*. Before calculating HP, HR, and HF1, we perform data augmentation on $C_{m}^{g}$ and $C_{m}^{p}$ to obtain ${\hat{C}}_{m}^{g}$ and ${\hat{C}}_{m}^{p}$, respectively. Specifically, the augmentation set includes all nodes in the original set and all ancestor nodes from the root of the hierarchical structure to these nodes. In this way, the closer the nodes are in the hierarchy, the more ancestor nodes they share. In other words, data augmentation for leaf nodes enables the classification error to be evaluated more highly if the classification result is more closely related to the ground-truth in the hierarchy. For example, if $C_{m}^{g}=\left\{H\right\}$ for the hierarchical structure in Figure 5, then ${\hat{C}}_{m}^{g}=\left\{A,C,F,H\right\}$. HP, HR, and HF1 are calculated by the following formulas:

(13) $$\begin{array}{cc} \mathrm{HP} & =\frac{\sum_{m}^{}\left|{\hat{C}}_{m}^{g}\cap {\hat{C}}_{m}^{p}\right|}{\sum_{m}^{}\left|{\hat{C}}_{m}^{p}\right|}, \end{array}$$

(14) $$\begin{array}{cc} \mathrm{HR} & =\frac{\sum_{m}^{}\left|{\hat{C}}_{m}^{g}\cap {\hat{C}}_{m}^{p}\right|}{\sum_{m}^{}\left|{\hat{C}}_{m}^{g}\right|}, \end{array}$$

(15) $$\begin{array}{cc} \mathrm{HF}1 & =\frac{2\times \mathrm{HP}\times \mathrm{HR}}{\mathrm{HP}+\mathrm{HR}}. \end{array}$$

When measuring the precision, recall, and F1 score for each image, the label *c* is predicted as positive if the score $s^{c}$ calculated in Section 3.3 is greater than 0.5.

Additionally, we adopted the average precision (AP) and the mean average precision (mAP) that were often used in multi-label classification tasks [14]. We calculate AP and mAP as follows:

(16) $$\begin{array}{cc} \mathrm{AP}(y_{c}) & =\frac{1}{L_{y_{c}}}\sum_{n=1}^{N}P_{ry_{c}}\left(n\right)\times \left(R_{ry_{c}}\left(n\right)-R_{ry_{c}}\left(n-1\right)\right), \end{array}$$

(17) $$\begin{array}{cc} \mathrm{mAP} & =\frac{1}{C}\sum_{c=1}^{C}\mathrm{AP}\left(y_{c}\right), \end{array}$$

where $L_{y_{c}}$ is the number of images relevant to the label $y_{c}$, *N* is the total number of retrieved images for the label $y_{c}$, *n* is the rank in the list of retrieved images, $P_{ry_{c}}\left(n\right)$ and $R_{ry_{c}}\left(n\right)$ are the precision and recall at the rank *n*. After sorting the scores *s* in descending order, the mAP can be calculated.

Generally, the average overall F1 (OF1), average per-class F1 (CF1), hierarchical F1 score (HF1), and mAP are considered the most important metrics for evaluating performance.

### 4.4. Experimental Results and Discussions

Table 1, Table 2, Table 3 and Table 4 show the quantitative experimental results of the proposed method and the comparison methods of the four datasets mentioned in Section 4.1, respectively. The experimental results show that the proposed method outperforms the other comparison methods overall in the multi-label attribute classification task for anime illustrations, which reflects the generalization of the proposed method across multiple datasets. In addition, the experimental results show that our GCN, which considers hierarchical relationships of labels, achieves higher performance than baseline networks (such as ResNet-101 [43]) that create the same latent space as ours and state-of-the-art GCN-based methods, such as P-GCN [38]. Furthermore, the proposed method shows higher HP, HR, and HF1 than the comparison methods in experiments conducted on various datasets. This confirms that the proposed method can more appropriately search for valuable relationships between labels in the hierarchical structure. In summary, the results confirm the effectiveness of considering the hierarchical structure of attribute information in multi-label classification tasks. In addition, Table 5 shows the computational time and space consumption of the proposed method and the comparison methods. Specifically, we select several GCN-based methods with high overall accuracy in the quantitative evaluation as comparative methods, and calculate their floating point operations (FLOPs) as the measure of time complexity, and the memory access cost (MAC) as the measure of space complexity, respectively. From this table, we can see that the proposed method exhibits similar or less computational time consumption while maintaining a space complexity closer to that of the comparison methods. In other words, the proposed method achieves better classification performance while maintaining similar or lower computational time and space consumption as compared to the previous methods.

**Figure 6:**
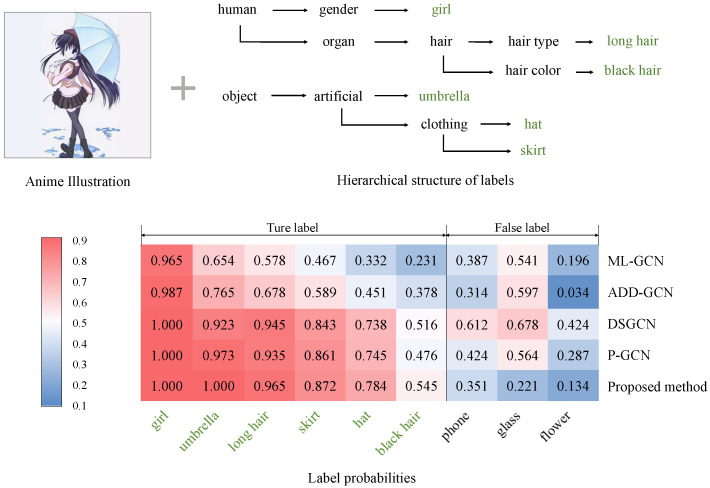
The heatmap displays the prediction scores of labels for an anime illustration from the Safebooru dataset [36]. The darkest red indicates the highest score, and the darkest blue indicates the lowest. We also show part of the hierarchical structure where the ground truth labels of the illustration are located and mark these labels in green font.

**Figure 7:**
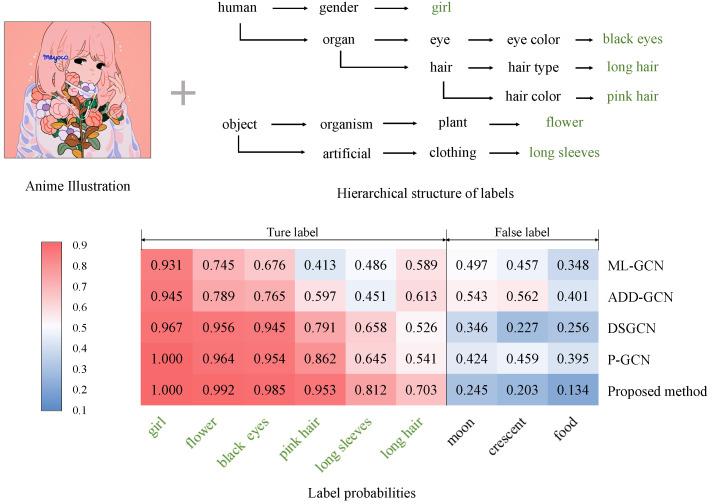
The heatmap displays the prediction scores of labels for an anime illustration from the DAF:re dataset [40]. The darkest red indicates the highest score, and the darkest blue indicates the lowest. We also show part of the hierarchical structure where the ground truth labels of the illustration are located and mark these labels in green font.

**Figure 8:**
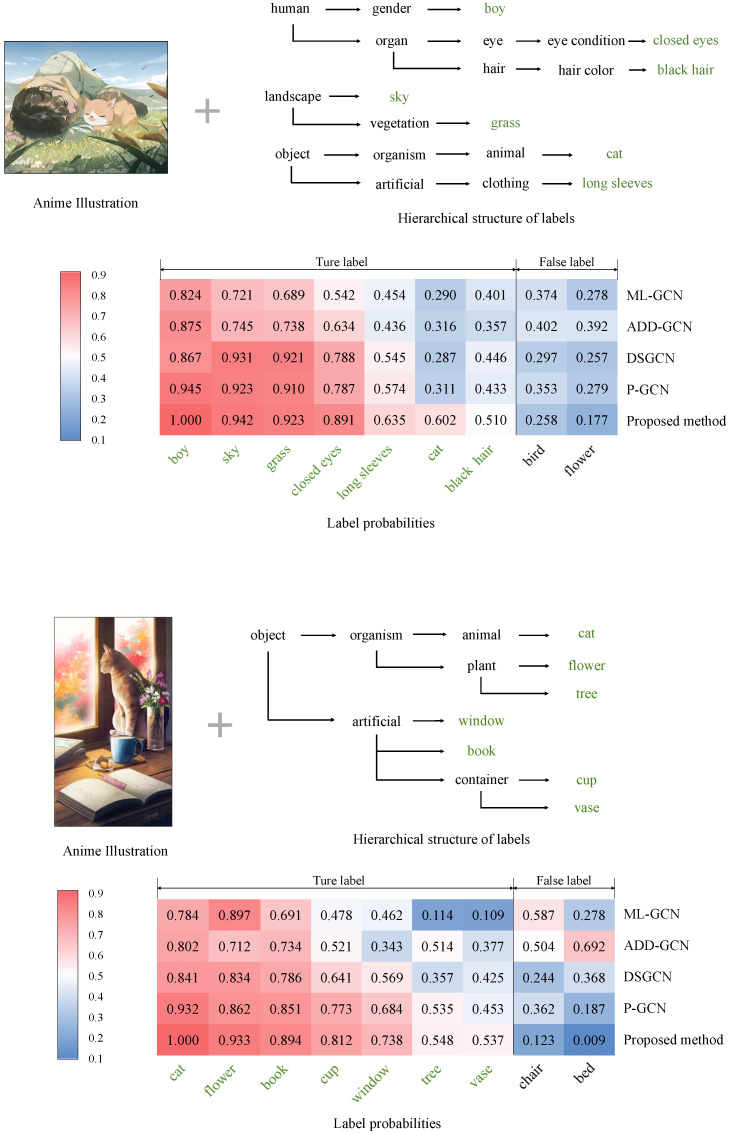
The heatmaps display the prediction scores of labels for two anime illustrations from the FG-BG dataset [41]. The darkest red indicates the highest score, and the darkest blue indicates the lowest. We also show part of the hierarchical structure where the ground truth labels of the illustrations are located and mark these labels in green font.

We also perform qualitative evaluations to demonstrate the effectiveness of our method. Specifically, we demonstrate some examples in the experiments in Figure 6, Figure 7 and Figure 8. In these figures, we show examples of the experiments on the Safebooru [36], DAF:re [40], and FG-BG [41] datasets, respectively. To control variables, we compare the performance of our method with comparison methods based on GCN. To visualize the specific classification performance of these methods, we draw heat maps consisting of the prediction scores of labels for each method. The *x*-axis of the heatmaps represents the top nine predicted leaf labels by various GCN-based methods on average, and the *y*-axis of the heatmaps represents our proposed method and four GCN-based comparison methods mentioned in Section 4.3: ML-GCN [11], ADD-GCN [14], DSGCN [39], and P-GCN [38]. In addition to the heatmaps, we also show part of the hierarchical structure where the ground truth labels of the illustration are located for a clear indication of how the consideration of the hierarchical relationship of the labels in the proposed method affects the final classification results. In the examples presented in Figure 6, Figure 7 and Figure 8, the predicted scores of the true labels are increased and those of the false labels are decreased overall after introducing the hierarchical structure. Specifically, in the example shown in Figure 6, the label *black hair*, which is incorrectly classified as negative by most of the other comparison methods, is correctly classified as positive after introducing the hierarchical structure because of the increase in the score of *long hair*, which has a close relationship with *black hair* in the hierarchical structure. In addition, the proposed method shows better classification accuracy than other methods for general anime character illustrations (Figure 6), illustrations that focus on character facial features (Figure 7), illustrations with complex backgrounds, and illustrations with no characters (Figure 8), which confirms its high versatility in classifying different styles and types of anime illustrations. Therefore, the effectiveness of our method is verified.

## 5. Conclusions

In this paper, we proposed a new hierarchical multi-label attribute classification model for anime illustrations using GCN. As existing multi-label classification models fail to consider the hierarchical relationship of attributes in images, we use hierarchical clustering to organize attribute information of anime illustrations into a hierarchical feature via hierarchical label assignments. This feature is used to construct a GCN-based classification framework that captures more comprehensive relationships between attributes from their co-occurrences. Our proposed approach outperforms previous methods, including the state-of-the-art, on multiple datasets, demonstrating excellent scalability and effectiveness. However, we acknowledge that our study only considers the most frequent labels and does not evaluate the classification accuracy for labels with lower frequencies. Moreover, it is still uncertain whether the manually constructed hierarchical structure of labels, based on predetermined rules, is the best structure for the images in the anime illustration datasets. In addition, we did not verify the impact of varying proportions of training data on the final classification results. In future work, we will perform these experiments further.

## Figures and Tables

**Table 1:** Performance comparison between our model and other image classification models on the Safebooru dataset [36]. We mark the best results in bold.

Method	OP	OR	OF1	CP	CR	CF1	HP	HR	HF1	mAP
ResNet-101 [43]	61.0	56.5	59.3	60.4	55.2	58.1	59.1	40.8	48.3	60.4
SSGRL [45]	69.0	57.2	64.2	70.2	58.2	61.3	60.9	47.2	53.2	68.6
DAN [12]	64.9	51.0	58.1	66.5	56.8	61.2	51.0	38.8	44.1	64.6
ML-GCN [11]	63.8	60.5	62.4	60.1	54.2	58.0	60.8	52.1	56.1	62.3
ADD-GCN [14]	69.6	64.1	67.2	66.4	**60.2**	62.0	61.7	52.8	56.9	68.1
DSGCN [39]	73.1	66.8	70.2	69.9	57.1	**66.3**	61.4	59.8	60.6	71.1
P-GCN [38]	69.1	58.9	63.1	**72.8**	58.9	64.2	65.3	58.8	61.9	70.2
**Ours**	**73.4**	**68.3**	**71.1**	69.8	56.4	65.9	**67.9**	**62.9**	**65.3**	**71.3**

**Table 2:** Performance comparison between our model and other image classification models on the DAF:re dataset [40]. We mark the best results in bold.

Method	OP	OR	OF1	CP	CR	CF1	HP	HR	HF1	mAP
ResNet-101 [43]	63.4	52.3	57.3	58.1	53.2	55.5	45.9	43.1	44.5	56.2
SSGRL [45]	69.1	54.6	61.0	64.8	54.8	59.4	51.0	42.3	46.2	60.1
DAN [12]	62.7	51.9	56.8	59.5	52.3	55.7	52.9	45.6	49.0	57.4
ML-GCN [11]	64.5	56.8	60.4	62.3	56.4	59.2	53.6	44.6	48.7	59.8
ADD-GCN [14]	65.2	54.0	59.1	61.9	54.4	57.9	55.0	47.4	50.9	58.9
DSGCN [39]	69.6	57.6	63.0	66.0	**58.1**	**61.8**	54.7	44.6	49.2	62.7
P-GCN [38]	67.7	**60.1**	63.7	64.3	56.5	60.1	57.1	49.2	52.9	62.0
**Ours**	**72.1**	59.7	**65.3**	**68.4**	56.1	61.7	**60.8**	**52.4**	**56.3**	**63.5**

**Table 3:** Performance comparison between our model and other image classification models on the FG-BG dataset [41]. We mark the best results in bold.

Method	OP	OR	OF1	CP	CR	CF1	HP	HR	HF1	mAP
ResNet-101 [43]	53.2	49.9	51.5	49.2	42.1	45.4	41.1	39.5	40.3	48.5
SSGRL [45]	60.2	51.3	55.4	56.5	51.9	54.1	45.6	37.3	41.0	54.6
DAN [12]	58.5	54.9	56.6	54.1	46.3	49.9	45.2	43.5	44.3	53.4
ML-GCN [11]	61.2	56.3	58.7	59.2	52.1	55.4	50.2	44.0	46.9	60.1
ADD-GCN [14]	62.6	58.7	60.6	57.9	49.6	53.4	48.4	46.5	47.4	57.1
DSGCN [39]	63.7	58.6	61.0	61.5	54.2	57.6	52.2	45.8	48.8	59.5
P-GCN [38]	**68.5**	56.5	61.9	**62.7**	57.6	60.0	49.6	46.5	51.9	60.7
**Ours**	67.9	**59.8**	**63.6**	62.5	**58.1**	**60.2**	**59.4**	**50.4**	**54.5**	**61.8**

**Table 4:** Performance comparison between our model and other image classification models on the iCartoonFace dataset [42]. We mark the best results in bold.

Method	OP	OR	OF1	CP	CR	CF1	HP	HR	HF1	mAP
ResNet-101 [43]	49.6	46.9	48.2	47.0	33.3	39.0	28.2	18.2	22.1	44.3
SSGRL [45]	51.4	50.0	50.7	51.5	39.3	44.6	30.3	19.3	23.6	46.1
DAN [12]	58.0	55.5	56.7	57.2	44.1	49.8	32.7	21.4	25.9	52.5
ML-GCN [11]	61.3	56.1	58.6	60.3	46.5	52.5	34.2	25.6	29.3	54.8
ADD-GCN [14]	60.5	54.8	57.5	59.1	45.3	51.3	35.7	25.1	29.5	53.6
DSGCN [39]	62.3	54.7	58.3	**61.7**	49.9	55.2	40.5	30.4	34.7	56.8
P-GCN [38]	63.9	57.8	60.7	59.9	**53.0**	56.2	48.9	34.1	40.2	58.7
**Ours**	**65.4**	**59.8**	**62.5**	60.9	52.4	**56.3**	**53.8**	**42.4**	**47.5**	**60.5**

**Table 5:** Comparison of computational time consumption (FLOPs) and space consumption (MAC) between our model and other image classification models.

Methods	FLOPs	MAC (byte)
ML-GCN [11]	5.21 G	101 M
ADD-GCN [14]	3.58 G	72.5 M
DSGCN [39]	3.71 G	77.6 M
P-GCN [38]	6.18 G	96.3 M
Ours	3.64 G	75.4 M

## Data Availability

A publicly available dataset was used in this work.
